# Practices and impact of biosecurity on pig performance in the West Region of Cameroon

**DOI:** 10.1186/s40813-026-00493-6

**Published:** 2026-03-05

**Authors:** Ronald Vougat Ngom, Zaverime Mafokemg, Emmanuel Assana

**Affiliations:** Department of Animal Production, School of Veterinary Medicine and Sciences, University of Ngaoundere, Ngaoundere, Cameroon

**Keywords:** Africa, Antibiotic, Antiparasitic, Biosecurity, Disinfectant, Production, Swine, Vaccine

## Abstract

**Background:**

In Africa, pig significantly contributes to animal protein supply to enhance food security. Pig farming in Cameroon is subject to perpetual health threats, with the spread of several diseases which strongly impact the production. This study aimed to evaluate the biosecurity compliance in pig farms in the West Region of Cameroon and assess its relationship with production performance. During on farm visit and face to face interview, data about biosecurity, veterinary drug usage and production parameters were collected in 78 randomly selected farms from April and October 2024.

**Results:**

Results showed that almost all of pig farms (98.7%) used antiparasitics and 94.9% used antibiotics for preventive (58.7%) or curative (41.3%) purposes. Vaccination against swine erysipelas was practiced by only 24.4% of farmers. More than half of the farms (64.1%) in the study area had a biosecurity score below 50 out of 100 and the average biosecurity score was 47 (ranged 28-66). Biosecurity measure related to personnel and visitors ranked the lowest score (21, range 0-71) while measures applied during farrowing and suckling period (77, range 20-100), and those associated with the environment and region (77, range 40-100) ranked the highest. The biosecurity level was significantly influenced by the years of experience and the training in pig farming of interviewees, the herd size, the type of breeds reared and the production type (farrower, grower‐finisher or farrow‐to‐finish). Findings revealed a moderate association between number of farrowings per sow per year and lactation length. A correlation was found between biosecurity and lactation length. The high mortality rate (44.64 ± 24.60%) recorded on farms with a poor level of biosecurity compared to others (15.45 ± 11.94%) shows the importance of biosecurity in the prevention and control of diseases and the improvement of production.

**Conclusions:**

The results of this study highlight the need for capacity building and awareness among farmers on the importance of implementing biosecurity measures for improving pig production.

## Background

Pig production plays an important role for the development of many African countries by providing affordable sources of meat as well as contributing to economy, through job creation and income generation [1, 2]. It is one of the avenues through which Africa can meet its need for animal protein because it shared the nutritional qualities of red and white meats. Indeed, in addition to its high biological value, it contains essential amino acids, and is easily digestible [2]. In comparison to other parts of the world, the projected demand for pig meat in some sub-Saharan countries would increase by 161% by 2050 [3]. This is associated with the significant increasing of the demand for animal-sourced food due to rising population, income, and urbanization.

Among the five regions in Africa, Central Africa is prominent in pig production, contributing to 17% of the total pig population of the continent [4]. With more than 4 million pigs, Cameroon is the largest pork producer in Central Africa [5]. Due to the importance of this sector, the Cameroonian Government through the Ministry of Livestock, Fisheries and Animal Industries has funded many projects to improve the productivity and reduce the importation of pork. Despite the sector's potential and the efforts made, recurring health and management issues remain the primary obstacles to its full development [6]. For example, the country is endemic for the African swine fever (ASF) since 1982 [7]. The ASF outbreak in 2014 and 2016 considerably reduced the size of the national herd and contributed to huge economic losses [8]. In addition to ASF, several infectious (swine erysipelas, neonatal diarrhoea, etc.) and parasitic diseases (gastrointestinal parasitosis, trypanosomiasis and strongylosis) were cited by Mouiche et al. [8] as important limiting factors of pig production in Cameroon.

To face the recurrence of diseases in pig farming, the use of veterinary drugs has become common practice for the treatment and prevention of diseases. In Cameroon, there are no prohibitions or controls on the use of antimicrobials for prophylactic and metaphylactic purposes in pig farms. The consumption trends of antimicrobials for veterinary use in food-producing animals between 2014 and 2019 showed the second highest mean quantity of antimicrobials adjusted by animal biomass in pigs (63 mg/kg) after poultry (213 mg/kg) [9]. Unfortunately, this widespread, sometimes extensive or inappropriate use of drugs has led to the emergence and alarming spread of antimicrobial resistance (AMR), a phenomenon that considerably compromises the effectiveness of these drugs for human and animal health [10]. Promoting a preventive approach with reducing antimicrobial usage such as biosecurity could be a better solution in this context. Moreover, a better biosecurity may help to improve productivity [11], farm profitability and animal welfare.

Biosecurity refers to all preventive and regulatory measures aimed at reducing the risks of introduction (external biosecurity) and spreading (internal biosecurity) of pathogens either between or within farms [12]. Biosecurity is therefore an essential tool in disease eradication and day-to-day animal health management programmes. In Cameroon, as in many African countries, data on biosecurity implementation in pig farming and their impact on production are lacking [13]. A single study by Kouam et al. [14]. only described biosecurity practices in pig farming in the Menoua division in the West region, one of the main pig production areas in Cameroon. This study aimed to assess the association between biosecurity and production performance in pig farms in West Region (Cameroon).

## Methods

### Study area and design

**Fig. 1:**
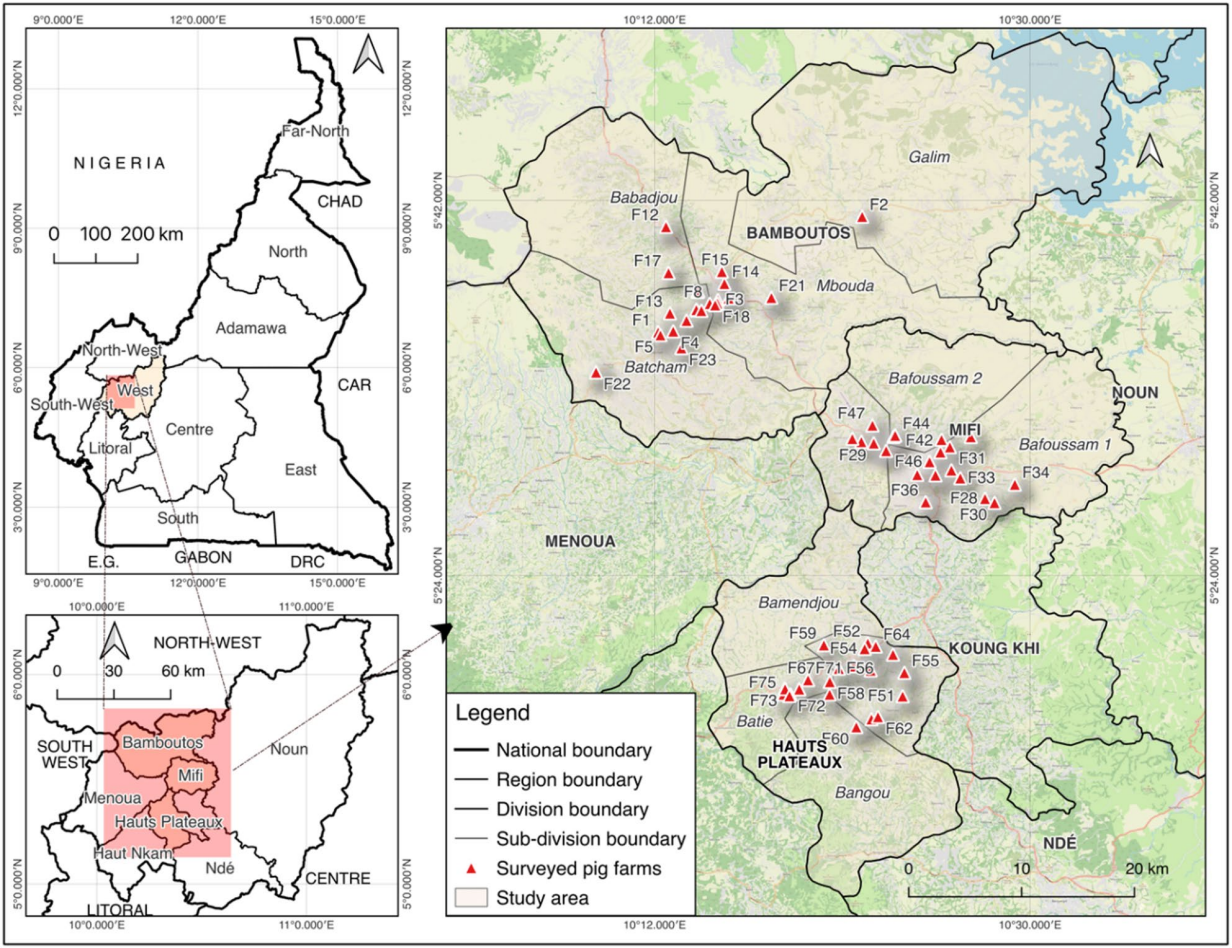
Distribution of the pig farms involved in the study in West Region of Cameroon

This study was carried out from April to October 2024 in the West region of Cameroon. The West region, with around 800,000 pigs, represents the second largest pig production area in Cameroon after the North-West [5]. Situated between latitudes 5°25’0‘’ and 5°35’0‘’ North and between longitudes 10°20’0‘’ and 10°35’0‘’ East, the West region covers an area of 13,892 km^2^. With a tropical sudano-guinean climate type, this region has an average rainfall of 1,365.8 mm and the temperatures fluctuated between 18 °C and 30 °C with an average of 25 °C. In 2022, the region’s population was estimated at 2 million [15]. Of the eight divisions of this region (Menoua, Nde, Noun, Koung-khi, Haut-Nkam, Haut Plateaux, Mifi and Bamboutos), three (Haut Plateaux, Mifi and Bamboutos) were selected for this study (Fig. 1). These three divisions have 60% of the pig produced in the region [16].

### Sample size and data collection

Sample size was estimated following the formula of Thrusfield [17] below:

$$\:N=\frac{\mathrm{1,96}^2\times\:\mathrm{P}(1-\mathrm{P})}{\mathrm{d}^2}$$

where N represents the sample size, P, the proportion of pig farms with a good level of biosecurity (4.73%) obtained by Kouam et al. [14]. in the Menoua division. After calculation, the sample size was estimated at 69 farms. In total, 78 pig farms were randomly selected from the list provided by the services of the Ministry of Livestock of the West Region. Only farms with at least 10 pigs and at least two years of existence were included in this study.

For this study, data were collected using a semi-structured questionnaire. After receiving the owners’ or managers’ oral consent, data were collected by means of a face to face interview survey and on-site observations.

## Veterinary drugs used in pig farms

The various classes of veterinary drugs used in farms were recorded. Data collected included the different active substances used and the reasons for use (preventive or curative). For antiparasitics, the different active substances used were listed, as well as the frequency of annual deworming and compliance with prescribed doses. Three levels of parasite control were defined as described by Clarys [18]. Good deworming means farmers followed the treatment doses and dewormed adult pigs every month and piglets at weaning or at age of one month. Average deworming corresponds to not following antiparasitic doses during treatment, not deworming of piglets and deworming of adults happening (or not) sometime. Poor deworming means farmers followed the treatment doses and dewormed adults every six month and piglets sometime.

## Evaluation of pig performance

The reproductive performance parameters of at least three sows were collected during visit in each farm. These parameters included age of first fertile service, age at first farrowing, number of farrowings/sow/year, and age at weaning. In addition, the number of piglets born alive at the last farrowing, the number of stillborn piglets, the number of weaned piglets and the number of piglets that died after weaning was collected to calculate the mortality until weaning and post-weaning mortality rates.

Pre- and post-weaning mortality rate were respectively assessed using the following formula (1) and (2):

1 $$\begin{aligned}&\:\mathrm{M}\mathrm{o}\mathrm{r}\mathrm{t}\mathrm{a}\mathrm{l}\mathrm{i}\mathrm{t}\mathrm{y}\:\mathrm{u}\mathrm{n}\mathrm{t}\mathrm{i}\mathrm{l}\:\mathrm{w}\mathrm{e}\mathrm{a}\mathrm{n}\mathrm{i}\mathrm{n}\mathrm{g}\:\cr&=\frac{\mathrm{n}\mathrm{u}\mathrm{m}\mathrm{b}\mathrm{e}\mathrm{r}\:\mathrm{o}\mathrm{f}\:\mathrm{p}\mathrm{i}\mathrm{g}\mathrm{l}\mathrm{e}\mathrm{t}\:\mathrm{d}\mathrm{e}\mathrm{a}\mathrm{d}\:\mathrm{b}\mathrm{e}\mathrm{f}\mathrm{o}\mathrm{r}\mathrm{e}\:\mathrm{w}\mathrm{e}\mathrm{a}\mathrm{n}\mathrm{i}\mathrm{n}\mathrm{g}\times\:100}{\mathrm{N}\mathrm{u}\mathrm{m}\mathrm{b}\mathrm{e}\mathrm{r}\:\mathrm{o}\mathrm{f}\:\mathrm{p}\mathrm{i}\mathrm{g}\mathrm{l}\mathrm{e}\mathrm{t}\:\mathrm{b}\mathrm{o}\mathrm{r}\mathrm{n}\:\mathrm{a}\mathrm{l}\mathrm{i}\mathrm{v}\mathrm{e}} \end{aligned}$$

2 $$\begin{aligned}&\:\mathrm{P}\mathrm{o}\mathrm{s}\mathrm{t}-\mathrm{w}\mathrm{e}\mathrm{a}\mathrm{n}\mathrm{i}\mathrm{n}\mathrm{g}\:\mathrm{m}\mathrm{o}\mathrm{r}\mathrm{t}\mathrm{a}\mathrm{l}\mathrm{i}\mathrm{t}\mathrm{y}\:\mathrm{r}\mathrm{a}\mathrm{t}\mathrm{e}\:\cr&=\frac{\mathrm{n}\mathrm{u}\mathrm{m}\mathrm{b}\mathrm{e}\mathrm{r}\:\mathrm{o}\mathrm{f}\:\mathrm{p}\mathrm{i}\mathrm{g}\mathrm{l}\mathrm{e}\mathrm{t}\:\mathrm{d}\mathrm{e}\mathrm{a}\mathrm{d}\:\mathrm{a}\mathrm{f}\mathrm{t}\mathrm{e}\mathrm{r}\:\mathrm{w}\mathrm{e}\mathrm{a}\mathrm{n}\mathrm{i}\mathrm{n}\mathrm{g}\:\times\:100}{\mathrm{n}\mathrm{u}\mathrm{m}\mathrm{b}\mathrm{e}\mathrm{r}\:\mathrm{o}\mathrm{f}\:\mathrm{w}\mathrm{e}\mathrm{a}\mathrm{n}\mathrm{e}\mathrm{d}\:\mathrm{p}\mathrm{i}\mathrm{g}\mathrm{l}\mathrm{e}\mathrm{t}\:}\end{aligned}$$

### Biosecurity assessment

The biosecurity level of each farm was assessed using the Biocheck.UGent^TM^ tool. The Biocheck.UGent system is a risk-based scoring system for quantifying biosecurity on a farm. It does not take into account a specific disease, but rather addresses biosecurity in general and focuses on aspects common to the transmission of different types of infectious disease. The survey sheet consisted of questions divided into several sub-categories for internal and external biosecurity, and each sub-category comprises 2 to 19 questions. The answer to each question gives a score between zero (when the measure is not implemented at all or the least optimal answer is given) and one (when the measure is fully implemented). Depending on the importance of a biosecurity measure, the score per question is multiplied by a weighting factor. The final internal and external biosecurity score can range from 0, indicating a total absence of the biosecurity measures described, to 100, indicating full implementation of the measures described. The total biosecurity score is obtained by averaging the internal and external biosecurity scores [19]. Once the survey form had been completed in the field, the information was transferred to the online questionnaire (https://www.biochekgent.com) to obtain the biosecurity scores. Biosecurity score varied between 0 and 100.

### Statistical analysis

Data collected were analysed using SPSS software version 16.0. Descriptive statistics were used to outline the socio-professional profile of the farmers and the key characteristics of the farms. Antibiotic families were categorized according to WHO classification into four main groups [20]: important antimicrobials (IA), highly important antimicrobials (HIA), critically important antimicrobials (CIA), and highest priority critically important antimicrobials (HPCIA). After the normality test performed, ANOVA followed by Duncan’s multiple comparison test was used to compare more than two means, and Student’s t test to compare two means. Pearson’s correlation test was used to establish the relationship between two variables. The correlation was categorized as low when the correlation coefficient *r* ≤ 0.3, moderate when 0.3 < *r* ≤ 0.5, strong 0.5 ≤ *r* < 0.7 and very strong when *r* ≥ 0.7 [11]. Significance level was set at 0.05.

## Results

### Profile of pig farmers and characteristics of farms

A total of 78 pig farms were surveyed in the west region. The demographics of farmers are presented in Table 1. Most of them were male (94.9%), with a secondary level of education (62.8%) and aged between 36 and 55 years old (59.0%). In addition, the majority of farmers did not have pig farming as their main activity (69.2%) and had a maximum of 10 years’ in the domain (53.8%). Less than one fourth (24.4%) have received training in pig farming. The majority of farms visited were farrow-to-finish category (87.2%), followed by farrower (11.5%) and grower-finisher (1.3%). Most of them were small scale farms with a maximum of 50 pigs (65.4%), followed by medium (51–150) and large (> 150) representing 24.3% and 10.2% of farms, respectively. Crossed breeds of pigs were found in 56.4% of farms while 30.8% of farms had exotic breeds. Local breed was found in only 6.4% of farms visited.

**Table 1:** Demographics of pig farmers in the West region of Cameroon (*n* = 78)

Variables	Number (*n* = 78)	Percentage (%)
Educational level		
Primary	12	15.4
Secondary	49	62.8
Higher	17	21.8
Age (year)		
[17–35]	11	14.1
[36–55]	46	59.0
>55	21	26.9
Gender		
Female	4	5.1
Male	74	94.9
Pig farming as main activity		
Yes	24	30.8
No	54	69.2
Received training on pig farming		
Yes	19	24.4
No	59	75.6
Years of experience in pig farming		
[0–10]	42	53.8
[11–20]	25	32.1
>20	11	14.1

### Veterinary drugs used in pig farms

Antiparasitics (98.7%), antibiotics (94.9%), vitamins (94.9%) and minerals (94.9%) were used in almost all the pig farms surveyed. However, vaccines, mainly against swine erysipelas (24.4%), hormones (12.7%) and anti-inflammatories (11.4%) were used on less than a quarter of them.

#### Antiparasitics and disinfectants used on farms

All the farms used disinfectants. The most commonly used for routine disinfection were virunet (33.0%), sodium hypochlorite or commercial bleach (23.1%), cresyl (21.8%) and cypermetrin (10.3%). The other (e.g. delthamethrin, TH4, profil, chlorine, tripuricide, *formaldehyde*) were used in less than 10% of the farms. For antiparasitics, the main active substances used were Ivermectin (64.1%), levamisole (35.9%) and albendazole (19.2%). Parasite control standards were well practised in 44.9% of farms.

#### Antibiotics used by pig farmers

**Fig. 2:**
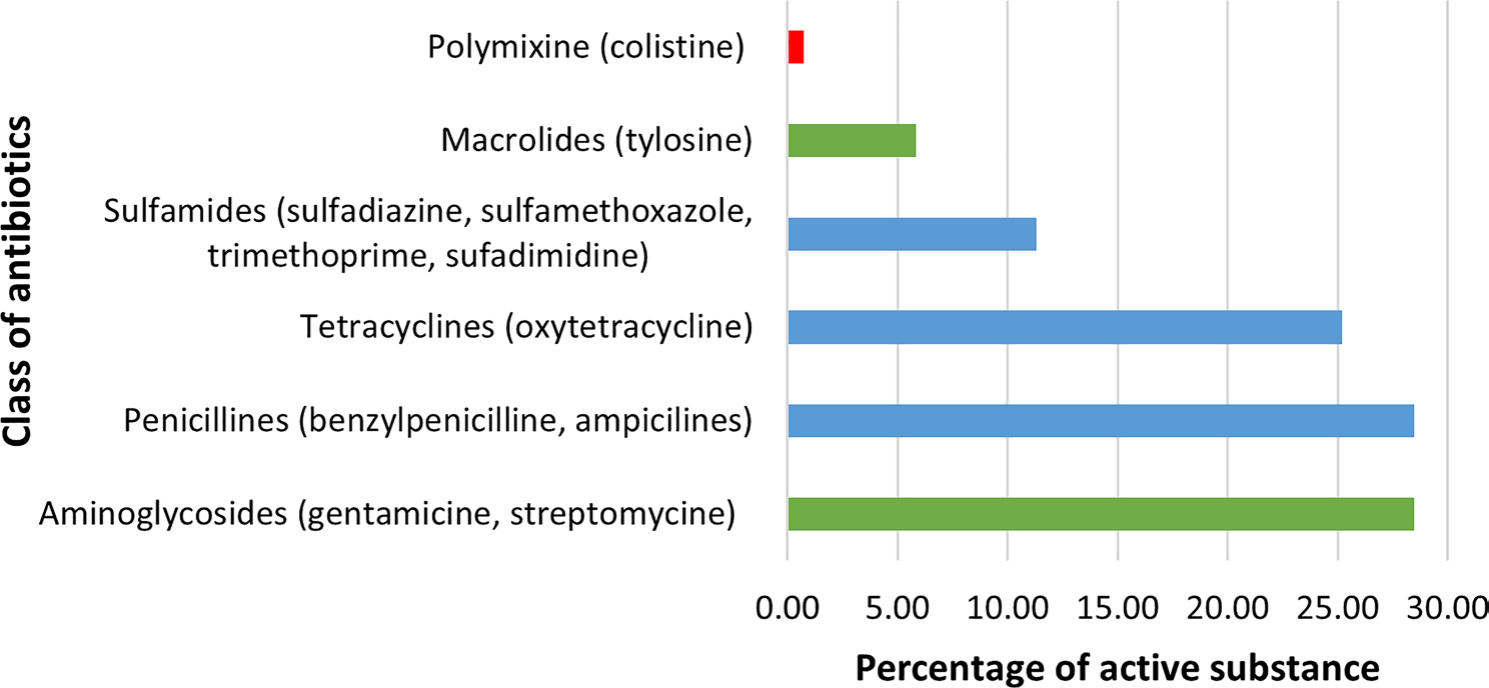
Frequency of antibiotics used per class in pig farms in West Region of Cameroon. Green are critically important antimicrobials. Bleu are highly important antimicrobials. Red are highest priority critically important antimicrobials

In the West region of Cameroon, antibiotics were used for both preventive and curative purpose and mostly administered to pigs by farmers (75.6%). Aminoglycosides (28.5%), penicillin (28.5%) and tetracyclines (25.2%) were the most commonly used antibiotic classes (Fig. 2). Oxytetracycline (25.2%) was the main active substance used. In general, 58.8% of antibiotics were used for disease prevention. The majority of CIA (64.9%) and HIA (56.2%) were used for preventive purpose while HPCIA were only used for treatment.

### Performance in pig farms visited

The pig breeds found in the studied farms were Duroc, Naima, Large white, Topic, Pietrain, landrace and crossed and local breeds. The average number of sows in the 78 herds was 10.9 (range 2-200) with an average of 2 boars (range 0–15) per farm. With 2 litters per sow per years, the average age of sows at first farrowing was 356 days (range 235–448). Piglets were weaned on average at 51.4 days (range 30–60). The overall mortality rate per farm was 32.2% while pre- and post-weaning mortality rates were 27.6% and 7.4%, respectively (Table 2). The post-weaning mortality rate was significantly higher (*p* < 0.001) on farms where deworming was not applied (8.4 ± 7.7%, *n* = 47) compared with the others (4.6 ± 3.9%, *n* = 21).

**Table 2:** Performance parameters in pig farms in West region of Cameroon (*n* = 78)

Variable	Average	SD	Min	Median	Max
Herd size	71.1	11.6	10	40	720
Number of sows/Herd	10.9		2	5	200
Number of boars/Herd	1.9		0	1	15
Age at first service (days)	244.9	37.0	186	244	335
Age at first farrowing (days)	356.4	40.8	235	350	448
Weaning age (days)	51.4	8.3	30	45	60
Number of litters/sow/year	1.7	0.4	1	2	2
Mortality rate until weaning	27.6	21.7	0	15.4	100
Mortality rate after weaning	7.4	5.9	0	0	100
Total mortality rate	32.2	24.8	0	20	100

SD: standard deviation, min: minimum; Max: maximum

### Biosecurity implementation in pig farms

In total 64.1% of farms had a biosecurity score below 50 out of 100. The average biosecurity score in the studied farms was 47 (range 28–66) with a poor external (46, range 28–64) and internal (47, range 20–71) biosecurity implemented similarly. Of all the subcategories ‘personnel and visitors’ (21, range 0–71) and ‘disease management’ (27, range 0–100) ranked the lowest scores. ‘Farrowing and suckling period’ (77, range 20–100), and ‘environment and region’ (77, range 40–100) recorded the highest scores (Table 3).

**Table 3:** Biosecurity scores in pig farms in West region of Cameroon (*n* = 78)

Sub-category	Average	SD	Median	Min	Max
*External biosecurity*	*46*	*7*	*47*	*28*	*64*
Purchase of animals and semen	67	15	72	32	100
Transport of animals, removal of manure/dead animals	40	6	38	29	57
Feed, water and equipment supply	31	9	33	0	53
Personnel and visitors	21	16	24	0	71
Vermin and bird control	40	23	30	0	90
Environment and region	77	24	70	40	100
*Internal biosecurity*	*47*	*12*	*46*	*20*	*71*
Disease management	27	4	20	0	100
Farrowing and suckling period	77	13	71	20	100
Nursery unit management	67	22	71	36	86
Fattening unit management	67	12	93	7	100
Compartmentalization and use of equipment	28	9	25	18	57
Cleaning and disinfection	35	25	40	0	95
*Overall biosecurity score*	*47*	*9*	*46*	*28*	*66*

SD: standard deviation, min: minimum; Max: maximum

Table 4 shows the variation of the level of biosecurity according to farmer’ demographics and farm’ characteristics. Overall biosecurity score was significantly (*p* = 0.029) higher (50.40 ± 8.60 versus 45.50 ± 6.90) in the farms where the respondents have received training on pig farming. Furthermore, the level of biosecurity on the farm increased significantly with the number of years of experience of the farmers (*p* = 0.049) and the herd size (*p* = 0.045). Biosecurity score was statistically (*p* = 0.032) higher on farrow-to-finish farms (47.63 ± 8.40) than on other farms. A higher biosecurity score (53.6 ± 8.1) was recorded in farms with a higher herd size. Farms rearing exotic breeds of pigs recorded a significantly (*p* < 0.001) higher biosecurity score (52.0 ± 7.7) as compared to farms with crossed breed (42.9 ± 7.1).

**Table 4:** Biosecurity scores according to farmer’ demographics and farm characteristics (*n* = 78)

Variable	Mean biosecurity score	*P*-value
Educational level		0.318
Primary (*n* = 12)	44.20 ± 7.10^a^	
Secondary (*n* = 50)	46.52 ± 8.60^a^	
Higher (*n* = 16)	49.0 ± 8.90^a^	
Age (years)		0.093
[17–35] (*n* = 11)	41.85 ± 12.50^a^	
[36–55] (*n* = 46)	48.14 ± 7.90^a^	
>55 (*n* = 21)	44.80 ± 7.70^a^	
Gender		0.412
Male (*n* = 74)	46.86 ± 8.50^a^	
Female (*n* = 4)	43.25 ± 7.20^a^	
Pig farming as main activity		0.20
Yes (*n* = 24)	48.50 ± 8.50^a^	
No (*n* = 54)	45.80 ± 8.40^a^	
Received training on pig farming		0.029
Yes (*n* = 19)	50.40 ± 8.60^b^	
No (*n* = 59)	45.50 ± 6.90^a^	
Years of experience in pig farming		0.049
[0–10] (*n* = 42)	45.10 ± 9.0^a^	
[11–20] (*n* = 25)	46.90 ± 7.10^b^	
>20 (*n* = 11)	52.10 ± 7.50^c^	
Production type		0.032
Farrower (*n* = 9)	43.0 ± 8.40^b^	
Grower-finisher (*n* = 1)	39.88^a^	
Farrow-to‐finish (*n* = 68)	47.63 ± 8.40^c^	
Herd size		0.045
Small (10–50)	45.6 ± 7.7^a^	
Median (51–150)	46.6 ± 9.6^a^	
High (> 150)	53.6 ± 8.1^b^	
Breeds reared in farms		< 0.001
Crossed breed (*n* = 44)	42.9 ± 7.1^a^	
Exotic (*n* = 24)	52.0 ± 7.7^b^	
Local breed (*n* = 6)	48.0 ± 9.6^ab^	
Crossed + Exotic (*n* = 5)	54.0 ± 3.8^b^	

### Associations between farmer profiles, pig performance and biosecurity level

**Fig. 3:**
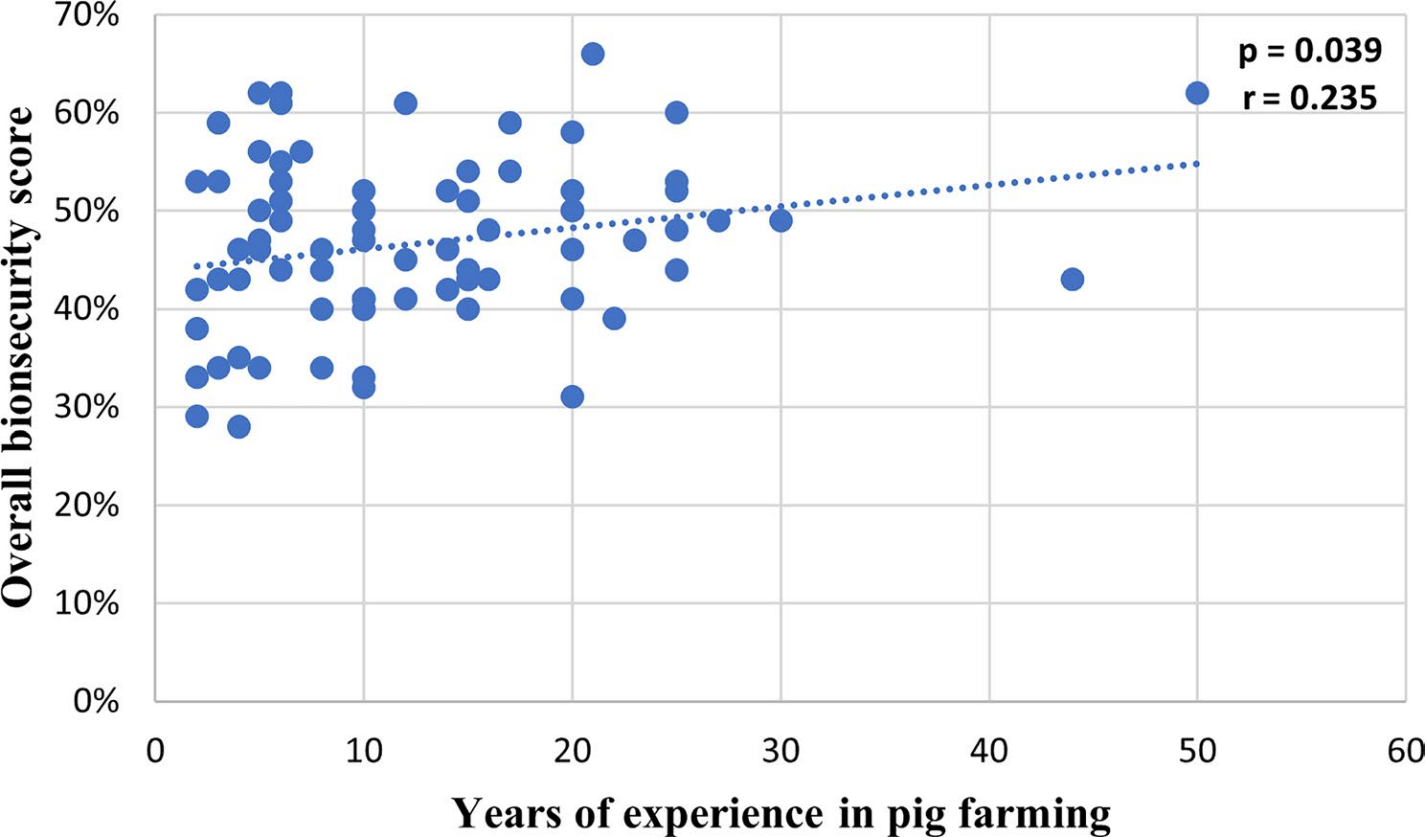
Correlation between years of experience of farmers and biosecurity score (*n* = 78)

Results showed a moderate association between the number of farrowings per sow per year and the lactation length (*r* = − 0.355, *p* < 0.01) and between the overall biosecurity score and the lactation length (*r* = − 0.482, *p* < 0.001). Similarly as presented in Fig. 3, a low significant correlation between the number of years of experience of the farmer and on-farm biosecurity level was found (*r* = 0.235, *p* = 0.039).

Regardless of the stage of production, the mortality rate was always significantly (*p* < 0.001) higher on farms with low biosecurity (average score below 50). The overall mortality rate was significantly (*p* < 0.001) higher in farms with poor biosecurity (44.6 ± 24.6% versus 15.4 ± 11.9%). The post-weaning mortality rate was 1.8 ± 1.2% on farms with biosecurity score above or equal to 50, whereas it was 11.2 ± 8.5% on farms with biosecurity below 50. Until weaning, the mortality rate on farms with poor biosecurity (average score below 50) was statistically (*p* < 0.001) higher (37.8 ± 22.1%) compare to other farms (14.0 ± 11.25%).

## Discussion

The main objective of this study was to evaluate the biosecurity compliance in pig farms in the West Region of Cameroon and assess it association with performance. During this study, several reproductive parameters were evaluated. The average age at first farrowing was 356 days. This result is similar to those obtained by Kouamo et al. [21]. in Douala (Littoral Cameroon) for the large white (342 days) and Landrace (354 days) breeds. However, it was higher than that obtained in local breed sows (338 days) in the town of Garoua in the North of Cameroon [22]. This variation could be due to breed and rearing conditions.

The average age at weaning recorded in this study was 51 days. A similar age was obtained in the Littoral region for the large white (57 days) and Landrace (56 days) breeds [21]. Simo et al. [23] reported that the weaning age of piglets is between 45 and 60 days in Cameroon. The weaning age in some European countries like Belgium (23.2 days) and Netherland (23.4 ays) are two times lower than our findings [24]. The average number of farrowings per sow per year was 1.75. This result correlates with findings of Ghomsi et al. [25] in three regions of Cameroon (1.90) (Far North, Centre, and West regions). All this results showed that more effort is needed to improve pig production in Cameroon.

The mortality rate until weaning recorded in the farms surveyed was 27.6%. These fairly high mortalities can be explained by several factors, the most commonly cited by farmers being: crushing, cannibalism, neonatal diarrhea, iron deficiency anaemia, primary or and secondary agalactia. Crushing and cannibalism are generally linked to the very narrow farrowing crates [26 unpublished] but also the poor management at the farrowing crate. Highest mortality rate until weaning (51.6%) was recorded by Kouamo et al. [21] in Douala. The difference with our results could be associated with the low adaptability of exotic breeds to local conditions. Indeed compare to our research, the study conducted in Douala had significant more exotic pig breeds.

After data analysing, it emerged that more than half of the farms surveyed (64.1%) had a biosecurity score below 50 out of 100. This could be associated to the insufficient knowledge of farmers concerning biosecurity [27] and the low level of involvement of veterinarians in monitoring these farms. These gaps suggest that although the 35.9% of farmers maintain satisfactory biosecurity standards, there is still room for improvement. This percentage of farms is lower than that obtained in the Menoua division, where 73.7% of farms had a poor level of biosecurity [14]. This divergence may be due to the fact that the study conducted in the Menoua division, involved a large number of small farms (< 50 pigs). Similar like our results, several studies have shown that the level of biosecurity on farms increases with the number of animals reared [19, 28]. Furthermore, the Menoua division was dominated by extensive farms (73%), which are characterized by minimal or non-existent biosecurity [14, 29]. The average overall biosecurity score for all farms surveyed was 47 out of 100 indicating low adherence to biosecurity practices aimed at minimizing disease risks and safeguarding livestock health. This could be explained by the fact that the majority of farmers focus on the use of veterinary drugs for disease prevention in the detriment of the application of biosecurity measures. This explains the high percentage of farms using antimicrobials in the study area. This result highlights the need to raise awareness among farmers in the study region about the need to implement biosecurity measures. The average biosecurity score is lower than that obtained in the Menoua (50) by Kouam et al. [14]. This difference could be due to the robustness of the biocheck tool used to assess biosecurity in our study.

The biosecurity measures least implemented in the study area were related to the subcategory ‘personnel and visitors’ and ‘disease management’. Indeed, none of the farms surveyed had a hygiene lock, a compulsory passage that allows anyone entering the farm to wash and disinfect their hands, and to put on the farm’s specific clothing and boots. The majority of farmers were unaware of this aspect, and a minority had a foot bath at the entrance to the farm, which for the most part were not functional. For some farmers, visits to their farms are strictly forbidden, but farm workers who have permanent access to the animals are not subjected to any disinfection process. This result explain the recurrence of diseases reported by farmers and the high mortality rate recorded. This finding is in agreement with Tona-Tona et al. [30] in the Democratic Republic of Congo, who presented these biosecurity measures as the weakness of all pig farms. The current context in several African countries where the level of knowledge and application of biosecurity measures is still precarious can justify this similarity [12].

The study’s findings revealed that the level of biosecurity was significantly higher (50.40 ± 8.60) in farms where participants had received training in pig farming. Farmers who had received training in pig farming were more aware of the importance of implementing biosecurity measures. This result is in line with that of Awoyomi et al. [28]., who showed that farmers’ level of knowledge was a major determinant of the application of biosecurity measures. Similarly, this study showed that the level of biosecurity increased significantly with the number of years of experience of the farmer. Farmers with more years of experience tended to better apply biosecurity measures because they knew the consequences of not complying with these measures [31]. In addition, biosecurity varied significantly with the type of farm, with the higher biosecurity score recorded in farrow-to‐finish farms. In this type of farm, farmers tend to apply biosecurity measures better, given the long stay of the animals on the farm, compared with other farms (breeder, fattener). Moreover, unlike fattening farms, these farms contain the young animals that are most susceptible to disease. Biosecurity level also varied with herd size, and the highest biosecurity score was recorded on farms with large herds. This result can be explained by the fact that large herds imply a greater investment by the farmer and therefore a greater risk in the event of disease. As a result, farmers are more careful about disease prevention practices. This result is in line with those described by Awoyomi et al. [28] in Nigeria.

At the end of the analyses, a moderate correlation was observed between the number of farrowings per sow per year and the average age at weaning. This result may reflect the ability of sows to return to heat rapidly as soon as they are separated from their litter, aligning with findings from studies in Ukraine indicating the reduction in the lactation period contributed to a reduction of the reproductive cycle of sow [32].

Another moderate association was established between biosecurity score and the average age at weaning. This correlation means that farmers with a good level of biosecurity were more likely to wean their piglets early. Indeed, a good biosecurity compliance leads to a rapid piglet’s growth, thus facilitating weaning. Pandofli et al. [33] showed that the number of farrowings per sow per year increased with the level of biosecurity. The number of farrowings per sow per year is directly linked to the age of piglets at weaning, which largely explains the link between biosecurity and the age of piglets at weaning.

The finding showed that the mortality rate varied significantly (p < 0.001) with the level of biosecurity at all stages of production. Poor biosecurity levels sustain the spread and transmission of infectious agents on farms [34, 35]. These infectious agents are at the root of several diseases that cause mortality at all stages of production. For example, neonatal diarrhea has been recorded as one of the main causes of piglet mortality in Cameroon [26]. These diarrhea may have a bacterial, or parasitic origin [36], and their prevention depends of biosecurity compliance and vaccination. Other diseases, such as erysipelas and ASF, are responsible for high mortality rates in pig farms in Cameroon, and their occurrence has been associated with poor application of biosecurity measures [7]. This corroborates Rodrigues da Costa et al. [37] and Putra et al. [38] who demonstrated that good biosecurity practices significantly reduced the incidence of mortalities. A recent review also showed the effectiveness of the implementation of biosecurity on preventing ASF in African pig farms [39]. A limitation of this study is related to the fact that only univariable analysis were performed. As for example, in addition to poor biosecurity, many other factors can influence the mortality rate recorded in farms. Future studies should collect other farm parameters and perform multivariable regression. 

## Conclusion

This study aimed to evaluate the biosecurity compliance in pig farms in the West Region of Cameroon and assess its relationship with pig performance. Results showed that biosecurity measures were poorly implemented in pig farms in the study region with pig farmers preferred antimicrobial usage rather than biosecurity for disease prevention. Furthermore, the mortality rate was strongly affected by the level of biosecurity on pig farms. Farms with poor level of biosecurity recorded higher mortality rates, hence the vital importance of biosecurity in pig farming in Cameroon. These results highlight the need to improve awareness and understanding of biosecurity implementation of pig farmers in Cameroon through effective communication, education, and training.

## Data Availability

The raw data supporting the conclusions of this article will be made available by the authors on request.

## Abbreviations

AMR: Antimicrobial resistance
ASF: African swine fever
CIA: Critically important antimicrobials
HIA: Highly important antimicrobials
HPCIA: Highest priority critically important antimicrobials
IA: Important antimicrobials
